# Levenberg-Marquardt Neural Network Algorithm for Degree of Arteriovenous Fistula Stenosis Classification Using a Dual Optical Photoplethysmography Sensor

**DOI:** 10.3390/s18072322

**Published:** 2018-07-17

**Authors:** Yi-Chun Du, Alphin Stephanus

**Affiliations:** Department of Electrical Engineering, Southern Taiwan University of Science and Technology, Tainan 71005, Taiwan; stevylib@yahoo.com

**Keywords:** arteriovenous fistula (AVF), hemodialysis (HD), dual PPG measurement node (DPMN), Levenberg-Marquardt algorithm (LMA)

## Abstract

This paper proposes a noninvasive dual optical photoplethysmography (PPG) sensor to classify the degree of arteriovenous fistula (AVF) stenosis in hemodialysis (HD) patients. Dual PPG measurement node (DPMN) becomes the primary tool in this work for detecting abnormal narrowing vessel simultaneously in multi-beds monitoring patients. The mean and variance of Rising Slope (RS) and Falling Slope (FS) values between before and after HD treatment was used as the major features to classify AVF stenosis. Multilayer perceptron neural networks (MLPN) training algorithms are implemented for this analysis, which are the Levenberg-Marquardt, Scaled Conjugate Gradient, and Resilient Back-propagation, to identify the degree of HD patient stenosis. Eleven patients were recruited with mean age of 77 ± 10.8 years for analysis. The experimental results indicated that the variance of RS in the HD hand between before and after treatment was significant difference statistically to stenosis (*p* < 0.05). Levenberg-Marquardt algorithm (LMA) was significantly outperforms the other training algorithm. The classification accuracy and precision reached 94.82% and 92.22% respectively, thus this technique has a potential contribution to the early identification of stenosis for a medical diagnostic support system.

## 1. Introduction

U.S. Renal Data System Annual Data Report (USRDS) recently claims, about 450 per million populations in Taiwan suffered by End-Stage Renal Disease (ESRD) and its incidence prevalence became most common among the highest in the world [1]. Taiwan Renal Registry Data System (TWRDS) confirmed the fact that, recently more than 80,000 Taiwanese patients are dialysis urgency and the count is growing [1,2].

Hemodialysis (HD) is a process of blood filtration of a patient with renal failure by using a device called Dialyzer. Before having HD treatment, the patient must go through a surgical access known as anastomosis, which returns the purified blood through a thin fiber after filtering out the waste fluid. The anastomosis is done a few weeks or months before HD treatment and it is done on patient’s elbow or wrist using the non-dominant arm [3,4,5]. There are three types of vascular access which are well known in HD treatment, namely Arteriovenous Fistula (AVF), Arteriovenous Graft and Venous Catheter respectively. AVF is the most recommended HD treatment. However, AVF should not be used for a longer time because it may cause stenosis which results in the dysfunctional fistula. 

Stenosis is an abnormal narrowing of the vessels caused by calcification processes or vascular wall thickening processes by new materials which may lead to abnormal blood pressure [6]. In the HD process, the recommended blood flow for arteriovenous fistula is more than 600 mL/min and more than 400 mL/min for arteriovenous graft [7,8]. When there is insufficient blood flow due to the narrowing of vessels then the remedial process on the fistula is performed. Therefore, it is important to monitor the treatment on the regular basis, to protect it from AVF stenosis, thus having total occlusion and extended fistula life.

In the case of HD treatments, access stenosis and vessel wall elasticity impair are frequently occurs [9].Vascular compliance was significantly associated with intra flow, resistance and changes in blood volume. Deformation of each PPG pulse shape was important parameter to classify the degree of stenosis (DOS) in HD patients. In the previous study, the bilateral difference in PPG pulse shape between two hands was also associated with the stenosis [7,8,9,10,11,12,13,14].

In clinical physical examination, angiography and Doppler ultrasound are commonly used for AVF monitoring [6]. Angiography is a golden standard for clinical vascular evaluation but it requires surgery and it is invasive [15]. Doppler ultrasound is an alternative way to evaluate the diameters of an AVF. Both of this clinical equipment are expensive and require experienced operators with the high knowledge to operate those [16]. On the other hand, as a low-cost measurement, PPG was developed for experimental purposes in vascular disease and its signal obtained from a noninvasive optical technique [17,18,19,20]. Although both golden standard aforementioned could provide high accuracy assessment, utilizing PPG signal might be a proper alternative solution to help patients for monitoring their arterial initial status [21].

Artificial neural network (ANN) based on multilayer perceptron is one of the techniques within the scope of pattern classification that is attracting the interest of researchers in recent years [22,23,24]. Currently, achieving precise goals in diagnosis and treatment is a very challenging issue to solve in clinical decision making. The appropriate techniques are required and the proper use of data is needed to solve this issue which corresponds to the pattern recognition problems. ANN has been used in the medical fields as a diagnosis of disease tool that uses principles in pattern classification [25]. Based on this fact, ANN has enough potential to make predictions in medical outcome such as arteriovenous fistula stenosis.

The proposed study uses multilayer perceptron neural network (MLPN) as classification technique. To set network model training algorithm were used such as Levenberg-Marquardt, scaled conjugate gradient and resilient back-propagation with respect to their weights and parameters. The performance of MLPN was influence by the neural network model structure and data selection [26]. Furthermore, to develop a reliable and robust network requires proper selection of appropriate data (feature) input. Therefore, the T-test is used as a feature selection technique to identify the presence of stenosis in HD patients. The PPG signal measured before and after HD treatment on the both left and right hand to calculate the values of RS and FS as features in time domains.

The proposed study was accomplished with an ANN model based on MLPN and tried to find a better training algorithm for dual PPG optical sensor system to have enough of potential for medical diagnosis decision making. The ANN stenosis classification model for HD patients used RS and FS value in the both hands before-after HD treatment as the input features. The sections in this paper are structured as follows: Section 2 describes the experimental detail with dual PPG measurement node and the techniques used to classify the degree of stenosis in a HD patient. Section 3 explains the experimental result. Finally, Section 4 provides the conclusions of the research.

## 2. Materials and Methods

### 2.1. Material

#### 2.1.1. Experimental Protocol

There are 11 subjects used in this study with a mean ± SD age of 77 ± 10.8. They were patients recruited from the Institutional Review Board (IRB): VGHKS17-CT3-11 of Kaohsiung Veterans General Hospital (KVGH). For this study, the patients relaxed for 10 min in supine position in a room with temperature of 25 ± 1 °C. The procedure was carried out in the same room specifically for HD treatment [8].

#### 2.1.2. Signal Measurement

**Figure 1:**
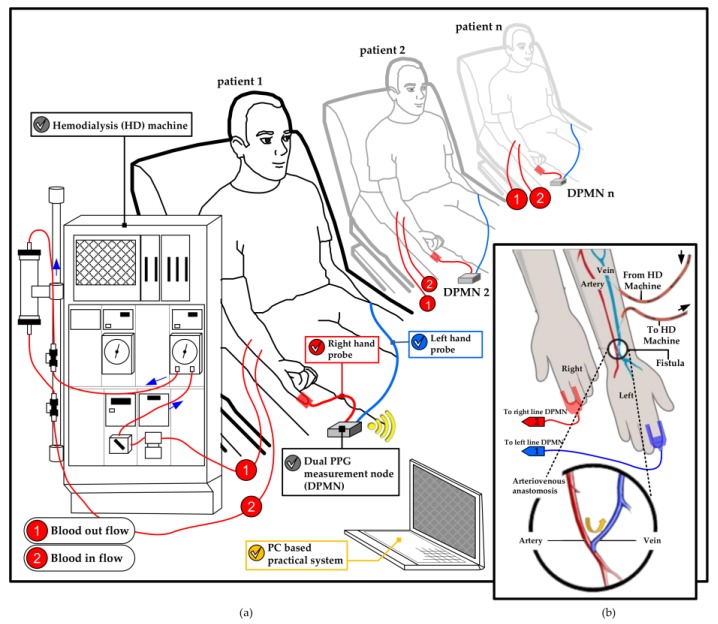
Dual optical PPG measurement technique: (**a**) PC Base practical PPG measurement with DPMN; (**b**) PPG probe placement and the fistula.

The changes in blood volume on the right and left hands were monitored in the supination position of forearms. The dual channel probes ware mounted within the index finger with a clip to acquire PPG signals from red infrared wavelengths reflection (680–940 nm) which synchronously collected at a sampling rate of 1 kHz. The collected PPG signal was then converted from analog to digital in an embedded system (MSP430, Texas Instruments, Dallas, TX, USA) to be transferred to a laptop using a blue-tooth module. This process runs on a set of devices called as the dual PPG measurement node (DPMN) which was given a power of 5-volt lithium battery. Next, the data was analyzed with MATLAB in a laptop. The PPG signal measurement technique explained above illustrated in Figure 1.

### 2.2. Methods

**Figure 2:**
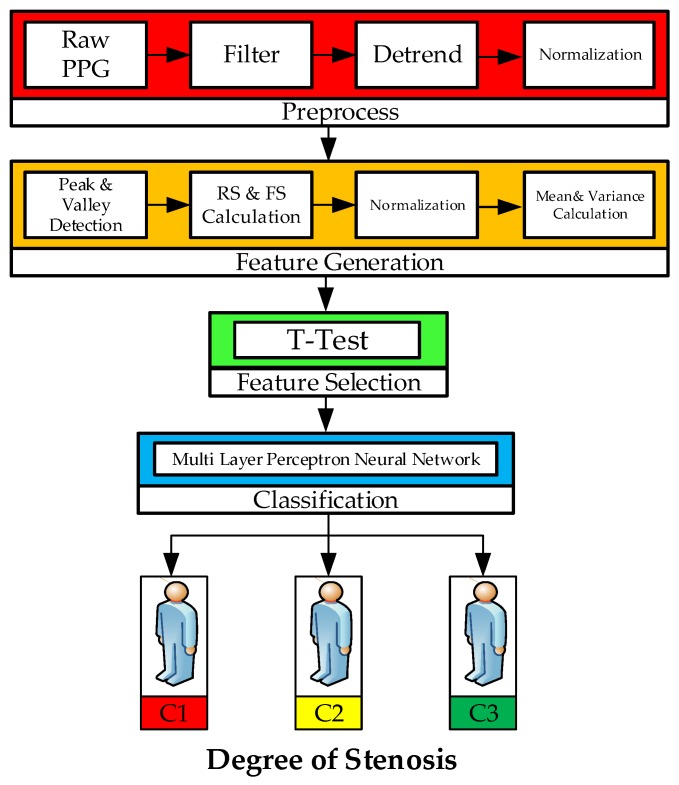
The technique used in the system development.

The main techniques of this study which are depicted in Figure 2 are outlined in four stages: (a) preprocessing of dual PPG signal, (b) feature generation (peak and valley detection, rising and falling slope calculation, normalization, mean and variance calculation), (c) feature selection (Statistical hypothesis T-test has been used for selecting the neural network input), (d) classification by utilizing MLP neural network to classify the input based on degrees of stenosis.

#### 2.2.1. Preprocessing of Dual PPG Signal

At this stage, the raw dual PPG signal was recorded from 11 patients and each segment of signal at least 1 min for analysis. In order to remove the noises caused by environmental signals, a moving average low pass filter (LPF) was employed with a robust local regression utilizing weighted linear least squares and a model of second degree polynomial (with a span of 1%).

#### 2.2.2. Feature Generation

Prior research by Allen and Murray [10,11,12] had experimented on the symmetric anatomical site of the human body by comparing two PPG signals called Bilateral PPG. The main goal was to extract the physiological features from the difference of bilateral PPG signals that was observed by signal shapes and temporal characteristics. Arts et al. [13] conducted a systematic quantitative investigation using the feature of [10] which was taken from the representative of leg stenosis patients and healthy subjects. In this study, feature generation was derived from the dual PPG signal extraction process observed from the signal shapes as investigated by [10,12]. The stages of feature generation begin from: peak and valley detection which was calculated from the values and locations of local maxima in selected PPG signal. The PPG signal has been de-trended to remove the outliers, offset and drifts before the calculation. The result then evaluated by the value of peak point (V_p_) minus the value from the valley’s starting point (V_n_) and then divided by the subtraction of the peak point location (T_pn_) with the location of the valley's starting point (T_n_) on each single signal PPG wave. This calculation was made to find the value of Rising Slope (RS) as described in Equation (1). Equation (2) used to calculate Falling Slope (FS) which is dealing with the next valley point (V_n+1_) on each single signal PPG wave and T_n+1_ as the next location of the valley’s point.

(1) $$\mathrm{Rising}\;\mathrm{Slope}\;\left(\mathrm{RS}\right)=\frac{V_{p}{\;-\;V}_{n}}{T_{\mathrm{pn}}{\;-\;T}_{n}}$$

(2) $$\;\mathrm{Falling}\;\mathrm{Slope}\;\left(\mathrm{FS}\right)=\frac{V_{n+1}{\;-\;V}_{p}}{T_{n+1}{\;-\;T}_{\mathrm{pn}}}$$

**Figure 3:**
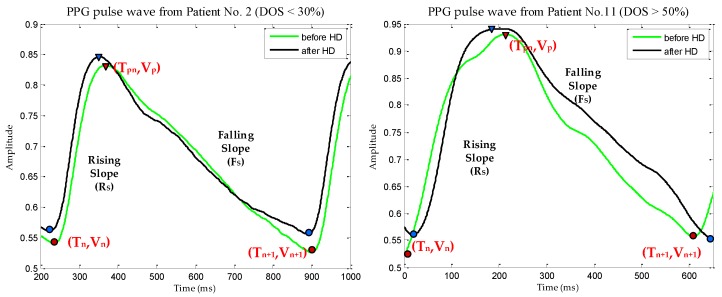
PPG pulse wave for RS and FS calculation before and after HD treatment in two patients.

The calculations were performed on the both right and left hand of the patients, before and after HD treatment. The detailed description of the term used in this study can be seen in the Figure 3. Normalization $x_{R,F}$ should be done to get the same range on each data (RS and FS) or known as rescaling [14,27]; in this study the data was rescaled from 0 to 1 utilizing Equation (3):

(3) $$\;x_{R,F}\;=\;\frac{x_{\mathrm{ik}}-\min\left(x_{k\;}\right)}{{\max(x}_{k})-\min\left(x_{k}\right)}$$

#### 2.2.3. Features Selection

The appropriate feature must be selected to improve prediction performance [28]. This stage begins with calculation of mean and variance using Equations (4) and (5), respectively, to provide the input on the statistical hypothesis used T-test in this study [27]:

(4) $${\bar{X}}_{b,a}\;=\;\sum \frac{x_{i}}{n}$$

(5) $$S_{b,a}^{2}\;=\;\frac{\sum_{i=1}^{n}(x_{i}-\bar{x}{)}^{2}}{n-1}$$

The last step in this stage was T-test calculation to get appropriate candidate feature which is shown from its statistical significance to the occurrence of stenosis in HD patients. T-test calculation is done by the following formula:

(6) $$t_{b,a}\;=\;\frac{\bar{x_{1}}\;-\;\bar{x_{2}}}{\sqrt{\left(\frac{S_{1}^{2}}{n_{1}}\;+\;\frac{S_{2}^{2}}{n_{2}}\right)}}$$

where, $\bar{x_{1}}$ is the mean of examined feature (RS or FS) before HD process and $\bar{x_{2}}$ is the mean of examined feature (RS or FS) before HD process. While, $S_{1}$ is the variance of examined feature before HD and $S_{2}$ is the variance of examined feature after HD. 

#### 2.2.4. Classification

(A) Degree of Stenosis (DOS)

Aforementioned specifically in this study, to classify AVF stenosis in HD patients the DOS is the main reference. The neural network as a classifier obtains input data from T-Test results performed on RS and FS features which is the parameter to identify stenosis occurrence in HD treatment. The detail DOS data correspond to the features used in this study is shown in Table 1.The selection of particular location for vascular access site in a hand was important with respect to the arterial anatomy and the adequacy of the venous [5]. Further, HD hand was a term to describe the selected location for HD treatment based on the recommendation from physicians or nephrologist, it usually in non-dominant side of the hands. 

These subjects in this study were distributed according to its severe class. In clinical research definition, the DOS was a degree index of narrowing normal vessel of arteriovenous fistula and it measured by B-mode ultrasound or angiography images. In the previous research [8,14,29] to grade the vascular disease, severe DOS was the main reference and the equation shows as below:

(7) $$\mathrm{DOS}\;\%=\;\left(1-\frac{d^{2}}{D^{2}}\right)\times 100\%$$

**Figure 4:**
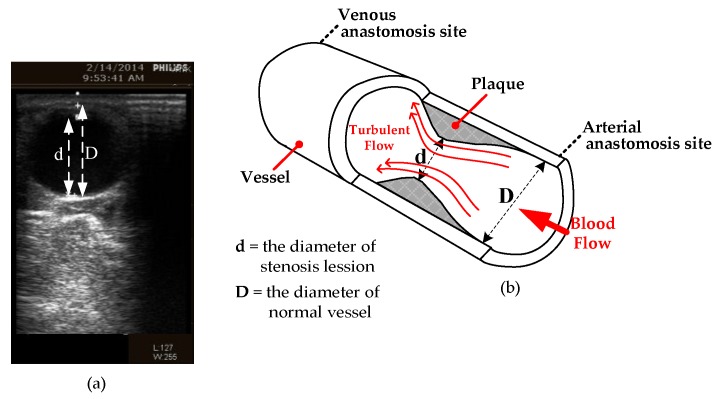
(**a**) B-mode ultrasound image of DOS measurement area; (**b**) the cross-section of the vascular access: the stenosis lesion diameter d and normal vessel diameter D [14].

In the blood flow direction, the stenosis lesion diameter represented by the value of “d” and the normal vessel diameter represent by “D”. The depiction of this term corresponds to the venous anastomosis and arterial anastomosis site are shown in Figure 4. 

The total occlusion occurs if the DOS is 100% while surgical treatment usually needed when over 50% and between 30% to 50% means during HD influences the efficiency. The distribution of three classes is designed based on the considerable amount of examination and the comment of a professional physician for the classification shown in Table 2.

(B) Multilayer Perceptron Neural Network

**Figure 5:**
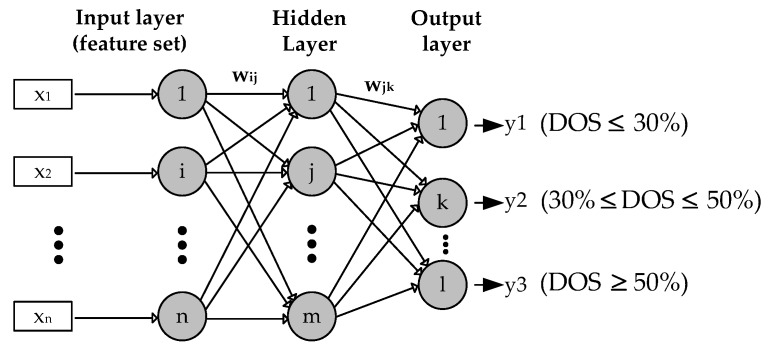
Multilayer Perceptron Network Structure.

Multilayer Perceptron (MLP) is the most common type used as feed forward Artificial Neural Network (ANN). Generally, MLP consists of one input layer, one hidden layer and one layer of computation output. Furthermore, to explain the definition of MLP used in this study can be illustrated as in Figure 5.

The input, hidden and output layer expressed in the index of neurons i, j and k respectively. The calculation of neurons in MLP is formulated in Equation (8), where r is the number of input data features, while x (x_1_, x_2_, …, x_n_) represents the feature value and w is the weight of the vector. In this study, the number of input data features r = 4, is taken from the variance and the mean value which is the selected feature by the T-test. The weights for connection from the input layer to the hidden layer are expressed in w_ij_ while the weights from the hidden layer to the k neuron in the output layer are expressed in w_jk_ (8) [27].

(8) $$\;v=\overset{r}{\underset{i=1}{\sum }}x_{i}.w_{i}$$

To generate the output signal, the value of v must be activated with the activation function. The Logistic Sigmoid or known as binary sigmoid was employed in this study to be the activation function. The error signal is propagated from the output layer to the hidden layer for the p iteration. The output value targeted to the k neuron and the real output obtained by the k neuron at the output layer [27]. In this study, the targeted output value is DOS ≤ 30% as class 1, 30% ≤ DOS ≤ 50% as class 2 and the last is DOS ≥ 50% as class 3. To update the weights on the connection between the hidden layers to the output layer is done by the following formula:

(9) $$w_{\mathrm{jk}}\left(p+1\right)=w_{\mathrm{jk}}\left(p\right)+\Delta w_{\mathrm{jk}}\left(p\right)$$

where $\Delta w_{\mathrm{jk}}\left(p\right)$ is the weight correction which is calculated using Equation (10):

(10) $$\Delta w_{\mathrm{jk}}\left(p\right)=\;\eta \times y_{j}\left(p\right)\times \delta_{k}\left(p\right)\;$$

where η is learning rate, while $\delta_{k}\left(p\right)$ is the gradient error in neuron k. The error gradient is determined from the derived activation function multiplied by the error in the output layer neuron. To calculate the error gradient corresponding to the activation function: Logistic Sigmoid (binary sigmoid) obtained from Equation (10):

(11) $$\;\delta_{k}\left(p\right)={\;y}_{k}\left(p\right)\times \left(1-y_{k}\left(p\right)\right)\times e_{k}\left(p\right)$$

where:

(12) $$\;y_{k}\left(p\right)=\frac{1}{1+e^{-v_{k}\left(p\right)}}$$

(C) MLPN Architecture

The architecture construction and the type of network were the main issue in ANN. Muzhou and Lee stated that adequate neural network architecture can improve the generalization of performance [30]. In this study, the optimal network architecture of the MLP model to be used initially has been determined as (4, 35, 3) after ten times of experiments. It means the dimensions of the layers are four input variables, 35 nodes are in the hidden layer and three output nodes, respectively. As previously described, the sigmoid logistics transfer function is used in the output layer and hidden layer.

(D) MLPN Training Algorithms

Three training algorithms were tested in this study to evaluate the ANN performance by 5-fold. However, not all algorithms are able to evaluate target output as desired. In this study, attempts were made to implement three training algorithms such as Levenberg-Marquardt, Scaled Conjugate Gradient, and Resilient Back-propagation. The detailed explanation related to the three training algorithms, is described as follows: 

(D-1) Levenberg-Marquardt Algorithm (LMA)

To solve nonlinear least squares problems, LMA is usually used as a standard algorithm. A combination of gradient descent and Gauss-Newton methods appears in this algorithm. In many cases, the LMA can guarantee problem-solving through its adaptive behavior [31]. If back-propagation (BP) is expressed as gradient descent, the algorithm becomes slow and doesn’t give an optimal solution [32]. On the other hand, if BP is expressed as Gauss-Newton, the algorithm has highest probability to give an optimal solution [33]. In this algorithm, the Hessian calculation approximation is shown in Equation (13) and the calculation of its gradient is expressed in Equation (14):

(13) $$H=J^{T}J$$

(14) $$g=J^{T}e$$

where Jacobian matrix represent by J and e indicate a vector of network error. And then the LMA behaves as Newton is expressed by the following equation:

(15) $$x_{k+1}=x_{k}-{\left[J^{T}J+\mu I\right]}^{-1}J^{T}e$$

where $x_{k+1}$ is a new weight that calculated as gradient function and current weight $x_{k}$ using Newton algorithm. 

(D-2) Scaled Conjugate Gradient Algorithm (SCGA)

The most popular iteration algorithm to solve the problem of large systems of linear equations is Conjugate Gradient. The iteration of the conjugate gradient is shown in Equation (16):

(16) $$x_{k}={\;x}_{k-1}+{\;\alpha }_{k}d_{k-1}$$

where k is the iteration index, α_k_ is the step length at k iteration, and d_k_ is the search direction. SCGA is the second order of Conjugate Gradient Algorithm which can minimize the purpose function on several variables. Moller [34] has the basis of algorithms which uses second-order techniques in second derivatives to find better local minimum solutions. SCGA uses step size scaling techniques to avoid consumption of learning iteration time. According to Moller, SCGA exhibits the superliner convergence on almost all problems as an advantage.

(D-3) Resilient Back-propagation Algorithm (RPA)

RPA is a learning algorithm that can improve the speed of convergence. RPA also minimizes the number of learning steps and different adaptive parameters using the Gradient Descent Algorithms (GDAs) principle to easily calculate learning schemes [35,36]:

(17) $$\Delta x_{k}=\;-\mathrm{sign}\left(\frac{\Delta E_{k}}{\Delta x_{k}}\right)\Delta k$$

The current weight vectors indicated by Δ$x_{k}$, $\Delta E_{k}$ is an error function at k and Δk is used to increase bias as formulated in (17).

(D-4) Training and testing time

In this study, training time is the time used by MATLAB to fetch the data input, do the training process and provides the classification result of MLP model developed. Testing time is detailed as the time used by the CPU to predict the class label of data input.

## 3. Results and Discussion

### 3.1. Technical Implementation

**Figure 6:**
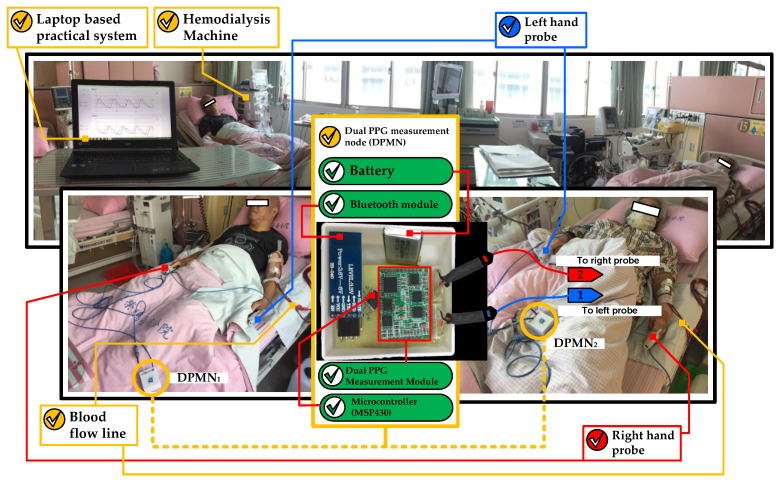
The dual optical PPG system implemented on the patients.

As explained in Section 2.1, this work was done based on our IRB to the HD patient that hospitalized in the hospital. Furthermore, the system has been implemented directly about the study with the technique as described in Section 2. Figure 6 shows the utilization of dual optical PPG sensors to classify the degrees of stenosis in HD treatment patients.

The use of DPMN in this study improves the quality of monitoring in multi-beds patients in the context of manpower availability. The laptop was used to collect patient data using several DPMN as depicted in Figure 6.

### 3.2. Result of Algorithm Implementation (Stage 1 to 3)

**Figure 7:**
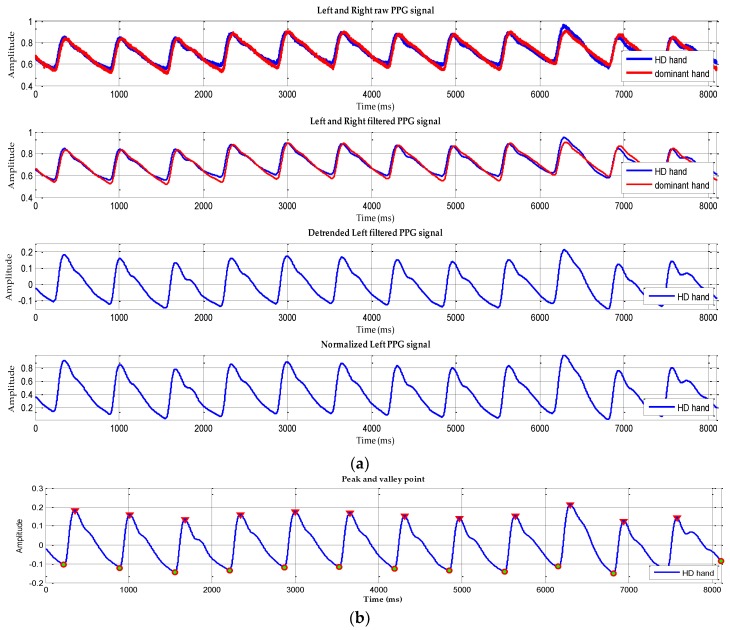
(**a**) The process data preprocessing; (**b**) Final result of peak and valley detection on selected twelve peak PPG signals.

In this work, MLPNs used Levenberg-Marquardt, Scaled Conjugate Gradient and Resilient Back-propagation as training algorithms were experimented to classify dual PPG signals obtained from HD treatment patients with degrees of stenosis. The comparison of these training algorithms was performed to obtain a good and efficient MLPN classifier. Feature selection is also very important to improve the classification performance quality [28] so that the results from the T-test are fed to the MLPN input layer. The experiments conducted in this study are divided into four stages as illustrated in Figure 2 and the result detailed as follows:

The first stage (preprocessing) previously explained have a result which is depicted in Figure 7a exhibits a raw PPG signal which has noise, offset and drift. To solve this problem, we performed the following steps: selecting 12 peaks of the raw input signal, filtering, de-trending and normalization respectively which is shown in four signal changes in Figure 7a sequentially.

Next, in Figure 7b shows filtered signal with peak and valley point detection and has undergone a de-trended process which calculated RS and FS. The peak and valley detection were applied to the selected twelve peaks of PPG signals, for each HD patient. The calculations of RS and FS were performed on the left and right processed PPG signals from the subjects recruited in this study, before and after HD treatment. This process is the final process in the feature generation stage. Then the results would be fed to the T-test as the selection stage to identify the stenotic feature in the HD patient.

In this study, the difference in deformation of PPG pulse shapes was compared to prove the occurrence of stenosis as described previously [7,8,13,14]. This physiological information was the expected feature to provide a proper input to produce optimal classification performance. 

As seen in Table 3, the third stage process generates results the T-test on RS features of the left hand which had statistical significance with the *p*-value < 0.05. It shows the feature RS of HD hand, before and after HD treatment, is appropriate input feature for classification. 

The statistical test proves the occurrence of stenosis after HD treatment process which was shown significantly in the left-hand as the HD hand. As described in Section 2.2.4, HD hand was the selected hand which is the location of the HD treatment done.Furthermore, this term will be used in the rest of the paper as it associated with the HD hand in which the PPG signal data is taken (as seen in Table 1).

### 3.3. Result of Stage 4

#### 3.3.1. Experimental Design and Training Algorithm selection

The basic parameters to acquire the robust and reliable MPLs, were obtained by try and error experiment which are shown in Table 4. Furthermore, a better training algorithm can be determined by setting all parameters of the same value and feed with different training algorithms afterward. There are three different developed MLP models, assessed by comparing its outputs with experimental results of training and testing data.

The training phase is a process in the network to find the input and output mapping by analyzing the training set repeatedly. In the training phase, the process of updating the weight based on the error information is being performed. As consequence, it runs slow or relatively takes more time. Table 5 displays one of the outcomes of the training phase with minimal errors (Mean Square Error—MSE) indicated by the accuracy of the MLP model predicting all desired target classes.

**Figure 8:**
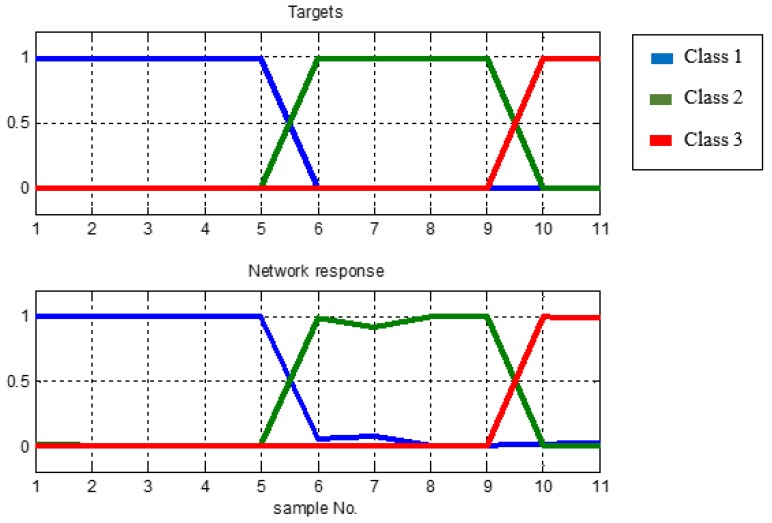
Network response to the targets class.

In the above table, it can also be concluded that the MLP model with the parameters used can classify the input data in accordance with the desired target classes that is, 5 target outputs of class 1, 4 of class 2 and 2 of class 3, respectively. Figure 8 illustrates the response of the network related to the class target to prove how well the MLP model is. Training curve is a curve that displays MSE versus iteration (Epoch). 

From the results of the experiments, the best performance was achieved at the 6th iteration with a value of 5.126 × 10^−4^ and is below the target value of pre-set target 1 × 10^−3^.

The testing phase is an advanced process of training phase where the MLP model is tested with a data that has never been known before to determine its performance. From the result shown in Table 6, the MLP model is very well recognized with newly learned data. It is proven that the new data was predicted correctly by MLP model that consists of four data from class 1, 1 of class 2 and 2 of class 3, respectively.

**Figure 9:**
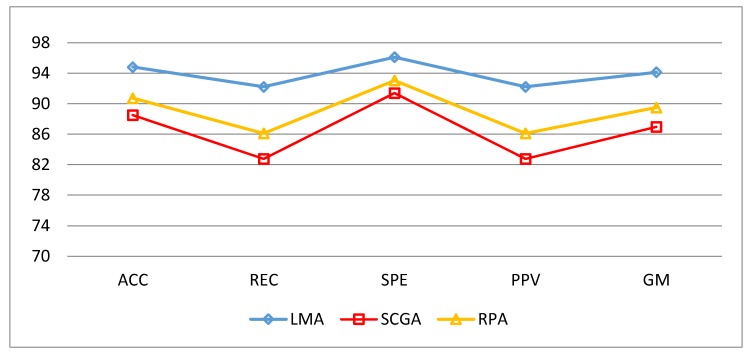
Five classification parameters of comparison in three algorithms.

The experiments carried out in this study first assessed the MLPN that was built using the LMA as its training algorithm, and the next was SCGA, and the last was RPA. Table 5 and Table 6 and Figure 8 and Figure 9 in this paper were taken from experimental results using LMA as training algorithm. Generally, the result of MLPN model using LMA shows excellent performance. With fixed setup parameters and unchanged architecture experiments performed also on SCGA and RPA, experiments are repeated for 10 times and the results can be seen in the next section.

#### 3.3.2. Performance Evaluation and Comparative Study of Training Algorithm

The performance of developed MLPN is analyzed using confusion matrix which is consisting of True Positive (TP), False Positive (FP), False Negative (FN) and True Negative (TN). The correctly predicted samples were represent by TP and TN, conversely the samples that were incorrectly predicted represent by FP and FN. Table 7 shows Confusion matrix for multi-class [14,37], and in this study there are 3 classes (DOS ≤ 30% = class 1, 30% ≤ DOS ≤ 50% = class 2, DOS ≥ 50% = class 3).

The values of TP, FP, FN and TN for the three classes are respectively calculated using Equations (18)–(21):

(18) $$\mathrm{TP}=C_{11}+C_{22}+C_{33}$$

(19) $$\mathrm{FP}=\left(C_{21}+C_{31}\right)+\left(C_{12}+C_{32}\right)+\left(C_{13}+C_{23}\right)$$

(20) $$\mathrm{FN}=\left(C_{12}+C_{13}\right)+\left(C_{21}+C_{23}\right)+\left(C_{31}+C_{32}\right)\;$$

(21) $$\mathrm{TN}=\left((C_{22}+C_{23}+C_{32}+C_{33}\right)+\left(C_{11}+C_{13}+C_{31}+C_{33}\right)\;+(C_{11}+C_{12}+C_{21}+C_{22}))$$

The calculations on the confusion matrix in this study uses the definitions C_11_, C_22_, C_33_ as MLPN models whose actual data and predictions have the same value. In other words, the model can predict the same as the target class. While in the first row, C_12_ should be class 1 (actual) but by the model predicted as class 2 and C_13_ should be class 1 (actual) but by the model predicted as class 3. For the next row, C_21_ should be class 2 (actual) but by the model was predicted as class 1 and C_23_ should be class 2 (actual) but by the model was predicted as class 3. For the last row, C_31_ should be class 3 (actual) but by the model was predicted as class 1 and C_32_ should be class 3 (actual) but by the model was predicted as class 2. By using confusion matrix, several performance indicators of a classifier can be calculated. To avoid or minimize the effects of the imbalance class in learning and evaluation process, this study combines different performance measures such as Accuracy (ACC), Sensitivity (REC), Specificity (SPE), Precision (PPV) and Geometric Mean (GM) [14,38]. To complete the information so that it can be conclude as the best MLPN performance then we added training time, test time, epoch of train and MSE in evaluation stage. As seen in Table 8 all training algorithm yield result above 80 percent. However, it is so clear that Levenberg-Marquardt Algorithm was superior to others. ACC = 94.82%, REC = 92.22%, SPE = 96.11%, PPV = 92.22%, GM = 94.13% were obtain using LM Algorithm as shown in Figure 9. The detailed classification performance result shown in Table 8.

As mentioned in C1, LM Algorithm could guarantee problem-solving through its adaptive behavior but it also slow algorithm which proved its TnT and TT were 1.167 and 0.228 s, respectively. Another indicator that strengthens the conclusions of LM Algorithm superiority was its high G-Mean value. The fastest algorithm was determined as RPA algorithm with the mean of TnT = 0.307 s and TT = 0.0055 s, respectively, so it is proved that RPA was a learning algorithm that could improve the speed of convergence. 

The evaluation performance of the classifiers had been built determined by the calculation of accuracy, sensitivity, specificity, precision and G-Mean respectively and Figure 9 shows the result comparison between the indicators.

The performance of neural network model in this experiment by using LMA provided good results toward unknown test data. This confirms that the over-fitting problem can be minimized by choosing network architecture sizes and features appropriately, as previous research by Guyon and Muzhou [28,30].

The results demonstrated that Multilayer Perceptron Neural Network with Levenberg-Marquardt was significantly outperforms other algorithm and succeeded to classify arteriovenous fistula stenosis in HD patients. Table 9 shows comparative table of similar research to identify AVF stenosis with proposed method predominance.

## 4. Conclusions and Future Work

In this study, we used Multilayer Perceptron Neural Network with Levenberg-Marquardt Algorithm to classify the degree of arteriovenous stenosis in a HD patient. As mentioned in the novelty of this study, from the three training algorithms could finally be recommended that MLPN with LM Algorithm could be used as a classification model to classify the degrees of arteriovenous fistula stenosis. The parameters and features used in this study strongly support the occurrence of good and efficient performance. It is proven from this work that the LM algorithm is a better back-propagation modification variant [39] although not too fast to converge over two other algorithms. In the previous study, that stenosis statistically significant has occurred on the HD hand [7,8]. Our design could not only evaluate the stenosis in HD hand, but also could analyze the bilateral difference between two hands referring previous studies methods [7,8,9,10,11,12,13,14]. Since the device of this system is low cost, efficiency and simple, we believe that the proposed study could provide potential assistive tools for DOS clinical evaluation, medical diagnostic support or commonly refer to as the Internet of Medical Things (IoMT). This study also has some limitations including the morphology of PPG signal is also influenced by cardiovascular regulation, blood pressure, patient age, arterial stiffness and other hemodynamic properties [7]. Furthermore, it could be varied by vasoactive drugs or endothelial function [40]. The clinical trial with a much larger sample set including a wide range of age, blood pressure, and other factors would be needed in the future research to verify the correlation between PPG shape with another arterial physiological information.

## Figures and Tables

**Table 1:** The data of HD patients.

No.	Class (Targets)	D	d	DOS (%)	Age	Gender		HD Hand	
						Male	Female	Right	Left
1	[1 0 0]	1.04	0.94	18.31	81	√			√
2	[1 0 0]	1.18	1.09	14.67	78	√			√
3	[1 0 0]	1.36	1.17	25.99	87	√			√
4	[1 0 0]	0.80	0.70	23.30	86	√			√
5	[1 0 0]	1.71	1.45	28.10	65	√			√
6	[0 1 0]	0.73	0.59	34.68	81	√			√
7	[0 1 0]	1.16	0.83	48.80	54	√			√
8	[0 1 0]	1.08	0.81	44.30	67	√			√
9	[0 1 0]	0.78	0.58	44.38	87	√			√
10	[0 0 1]	0.88	0.44	74.72	75		√		√
11	[0 0 1]	0.98	0.23	94.50	86	√			√

**Table 2:** Class partition based on DOS [14].

DOS	Class
DOS ≤ 30%	1
30% ≤ DOS ≤ 50%	2
DOS ≥ 50%	3

**Table 3:** RS and FS features of HD hand.

Feature	Conditions	Class 1					Class 2				Class 3		T-Test	Hypothesis
		No. 1	No. 2	No. 3	No. 4	No. 5	No. 6	No. 7	No. 8	No. 9	No.10	No.11		
**RS**	**before HD**	0.0792	0.0724	0.0867	0.0789	0.0854	0.1058	0.0751	0.1194	0.1194	0.1020	0.0874	0.0211	**Rejected**
	**after HD**	0.1139	0.0917	0.0865	0.0778	0.1023	0.1058	0.0958	0.1307	0.1243	0.1035	0.0867		
**FS**	**Before HD**	0.1233	0.0920	0.0847	0.1037	0.1572	0.0950	0.0871	0.0686	0.0686	0.0770	0.1146	0.5893	**Not Rejected**
	**after HD**	0.1348	0.1254	0.0714	0.0930	0.0937	0.0950	0.0757	0.1255	0.1254	0.1254	0.0790		

**Table 4:** Parameter setup.

Parameters	Setup Value (Configuration)
Learning rate (lr)	0.1
Error goal	0.001 (1 × 10^−3^)
Epoch	1000
momentum	0.95
Hidden layer activation function	Logistic Sigmoid (logsig)
Output layer activation function	Logistic Sigmoid (logsig)
Structure Dimensions	4, 35, 3
Training Algorithm	Levenberg-Marquardt (LMA)
	Scaled Conjugate Gradient (SCGA)
	Resilient Backpropagation (RBA)
Class 1 − (DOS ≤ 30%)	[1 0 0]
Class 2 − (30% ≤DOS ≤ 50%)	[0 1 0]
Class 3 − (DOS ≥ 50%)	[0 0 1]

**Table 5:** MLPN output.

Target Class	MLPN Output			MSE (10^−3^)
[1 0 0]	**0.9681**	0.0208	0.0046	0.0375
[1 0 0]	**0.9899**	0.0102	0.0099	0.0834
[1 0 0]	**0.9865**	0.0015	0.0126	0.0087
[1 0 0]	**0.9680**	0.0137	0.0101	0.0901
[1 0 0]	**0.9782**	0.0119	0.0079	0.0151
[0 1 0]	0.0160	**0.9824**	0.0052	0.0107
[0 1 0]	0.0564	**0.9662**	0.0021	0.1879
[0 1 0]	0.0250	**0.9946**	0.0011	0.0351
[0 1 0]	0.0106	**0.9957**	0.0094	0.0023
[0 0 1]	0.0509	0.0094	**0.9809**	0.0461
[0 0 1]	0.0435	0.0097	**0.9798**	0.0572

Note: The bold number shows the maxima of the MLPN output.

**Table 6:** Unknown data test output.

Target Class	MLPN Output		
[1 0 0]	**0.9932**	0.9611	4.08 × 10^−7^
[0 1 0]	0.0031	**0.8854**	0.0701
[1 0 0]	**1.0000**	0.0305	1.45 × 10^−5^
[1 0 0]	**0.9995**	0.2617	1.48 × 10^−5^
[1 0 0]	**1.0000**	0.0291	1.37 × 10^−7^
[0 0 1]	0.5613	0.0003	**0.8281**
[0 0 1]	0.9826	0.0004	**0.9931**

Note: The bold number shows the maxima of the MLPN output.

**Table 7:** Confusion Matrix of three classes.

		Predicted		
**Actual**	**Classes**	DOS ≤ 30%	30% ≤ DOS ≤ 50%	DOS ≥ 50%
	DOS ≤ 30%	C_11_	C_12_	C_13_
	30% ≤ DOS ≤ 50%	C_21_	C_22_	C_23_
	DOS ≥ 50%	C_31_	C_32_	C_33_

**Table 8:** Classification Performance Results.

Training Algorithm	* Performance								
	ACC (%)	REC (%)	SPE (%)	PPV (%)	GM (%)	Training Time (s) (TnT)	Test Time (s) (TT)	Epochs	MSE
Levenberg-Marquardt Algorithm (LMA)	94.816 (±5.00)	92.221 (±7.50)	96.11 (±3.75)	92.221 (±7.50)	94.126 (±5.00)	1.167 (±1.40)	0.228 (±0.18)	5.50 (±0.85)	0.082 (±0.22)
Scaled Conjugate Gradient Algorithm (SCGA)	88.52 (±4.08)	82.78 (±6.11)	91.39 (±3.06)	82.78 (±6.11)	86.96 (±4.67)	0.409 (±0.06)	0.062 (±0.01)	60.2 (±17.65)	0.003 (±3.3 × 10^−3^)
Resilient Back-propagation Algorithm (RPA)	90.74 (±3.15)	86.11 (±4.72)	93.06 (±2.36)	86.11 (±4.72)	89.51 (±4.72)	0.307 (±0.04)	0.055 (±27.54)	31.000 (±12.41)	0.003 (±0.01)

* Mean (± Standard Deviation).

**Table 9:** Comparative Table of Similar Research.

The Approaches	Hsien-Yi Wang et al. [3]	Jian-Xing Wu et al. [7]	Wei-Ling Chen et al. [15]	Du, Y.C., & Stephanus, A. [14]	Proposed Method
The clinical stenosis detector	Stethoscope	Doppler Ultrasound	Stethoscope	PPG	PPG
Classifier	RBF Neural Network	I-G Decision Making	ANFIS	ESVM-OVR	MLP Neural Network
The number of classes	2	2	3	3	3
Accuracy	-	-	-	90	94.82
System Performance rate—PPV	87.84	>80	-	91.67	92.22
GM	-	-	-	-	94.13
CPU time rate (training)	-	-	-	0.22	0.228

## Abbreviations

The following abbreviations are used in this manuscript:

PPG	Photoplethysmography
RS	Rising Slope
FS	Falling Slope
AVF	Arteriovenous Fistula
DOS	Degree of Stenosis
MLPN	Multilayer Perceptron Neural Network
USRDS	U.S. Renal Data System Annual Data Report
ESRD	End-Stage Renal Disease
TWRDS	Taiwan Renal Registry Data System
HD	Hemodialysis
LPF	Low pass filter
DPMN	Dual PPG measurement node
LMA	Levenberg-Marquardt Algortithm
SCGA	Scaled Conjugate Gradient Algorithm
RPA	Resilient Backpropagation Algorithm
ACC	Accuracy
REC	Recall
SPE	Specificity
PPV	Positive Predictive Value (Precision)
GM	Geometric Mean
TnT	Training Time
TT	Test Time
MSE	Mean Square Error
